# Variations in Mortality in Children Admitted with Pneumonia to Kenyan Hospitals

**DOI:** 10.1371/journal.pone.0047622

**Published:** 2012-11-05

**Authors:** Philip Ayieko, Emelda A. Okiro, Tansy Edwards, Rachel Nyamai, Mike English

**Affiliations:** 1 Health Services Research Group, Centre for Geographic Medicine Research - Coast, Kenya Medical Research Institute/Wellcome Trust Research Programme, Nairobi, Kenya; 2 Malaria Public Health and Epidemiology Group, Centre for Geographic Medicine Research - Coast, Kenya Medical Research Institute/Wellcome Trust Research Programme, Nairobi, Kenya; 3 MRC Tropical Epidemiology Group, London School of Hygiene and Tropical Medicine, London, United Kingdom; 4 Division of Paediatrics, Ministry of Medical Services, Nairobi, Kenya; 5 Nuffield Department of Medicine and Department of Paediatrics, University of Oxford, Oxford, United Kingdom; Boston University, United States of America

## Abstract

**Background:**

The existing case fatality estimates of inpatient childhood pneumonia in developing countries are largely from periods preceding routine use of conjugate vaccines for infant immunization and such primary studies rarely explore hospital variations in mortality. We analysed case fatality rates of children admitted to nine Kenyan hospitals with pneumonia during the era of routine infant immunization with Hib conjugate vaccine to determine if significant variations exist between hospitals.

**Methods:**

Pneumonia admissions and outcomes in paediatric wards are described using data collected over two time periods: a one-year period (2007–2008) in nine hospitals, and data from a 9.25-year period (1999-March 2008) in one of the participating hospitals. Hospital case fatality rates for inpatient pneumonia during 2007 to 2008 were modeled using a fixed effect binomial regression model with a logit link. Using an interrupted time series design, data from one hospital were analysed for trends in pneumonia mortality during the period between 1997 and March 2008.

**Results:**

Overall, 195 (5.9%) children admitted to all 9 hospitals with pneumonia from March 2007 to March 2008 died in hospital. After adjusting for child’s sex, comorbidity, and hospital effect, mortality was significantly associated with child’s age (p<0.001) and pneumonia severity (p<0.001). There was evidence of significant variations in mortality between hospitals (LR χ^2^ = 52.19; p<0.001). Pneumonia mortality remained stable in the periods before (trend −0.03, 95% CI −0.1 to 0.02) and after Hib introduction (trend 0.04, 95% CI −0.04 to 0.11).

**Conclusions:**

There are important variations in hospital-pneumonia case fatality in Kenya and these variations are not attributed to temporal changes. Such variations in mortality are not addressed by existing epidemiological models and need to be considered in allocating resources to improve child health.

## Introduction

Pneumonia is the leading cause of childhood deaths worldwide accounting for an estimated 1.8 million deaths annually. [1] A significant number of these deaths continue to occur in low income countries and within hospitals in these settings. [2] While there are summary and disease specific hospital death rates available for developing countries data quality may be poor. [3] There are in particular, significant gaps in understanding mortality, including childhood pneumonia mortality. [4] First, unlike in developed countries the potential variations in hospital pneumonia mortality have not been studied in any detail in Kenya or similar settings neither have potential causes of these variations been explored. [5], [6] Second, existing estimates mainly focus on geographical variations and few studies have investigated temporal variations in hospital death rates. In order to be convincing, investigations of temporal variations for pneumonia mortality must account for major policy interventions targeting disease specific mortality, for example introduction of pneumonia vaccines in routine use. During the past decade, two pneumonia vaccines have been introduced in routine use in Kenya: the *H. influenzae* Type B (Hib) vaccine in 2001 and, after the study now reported, pneumococcal conjugate vaccine in 2012. Data describing pneumonia outcomes in low-income countries (LICs) in the era after vaccine introduction are sparse.

One important application of data generated from studies investigating variations in pneumonia death rates would be in reviewing epidemiological models currently being used for decision making. The present decision making models assume that pneumonia outcomes are relatively homogeneous. For example an international analysis of pneumonia vaccination cost effectiveness including 72 developing countries was able to identify only four published studies on which to base its severe pneumonia case fatality rate. [7] Such simplifying assumptions are partly justified by lack of data but their impact needs to be examined. Better data on and understanding of pneumonia mortality are therefore important to improve models that help direct current and future resource use in health and as a means to understand health system performance and direct efforts to areas where performance is poor.

The purpose of the analysis presented here was to explore two scenarios in which variations in hospital pneumonia mortality rates could occur. Firstly using data collected simultaneously from nine hospitals we examined whether pneumonia mortality varies significantly by hospital. Outcomes were measured using vital information recorded in paediatric medical notes during a one-year period. Secondly, and using time series data collected in one hospital over a 9.25-year period, we conducted further analysis to identify evidence of temporal variation in pneumonia mortality within the hospital over time. Variations in death rates within hospital over time could be explained by several factors including major policy changes, changes in practice or organization of care. One such policy change implemented during the time period covered by our temporal analysis (1998 to March 2007) and relevant for pneumonia outcomes was introduction of Hib vaccine into routine use in Kenya in 2001. Using interrupted time series analysis we investigated pneumonia mortality over time while accounting for the introduction of Hib vaccine during the time horizon of our analysis.

## Methods

### Ethics Statement

During admission to Kilifi District Hospital written informed consent to enroll patients in ongoing studies is obtained from parents or guardians of paediatric admissions. In the remaining eight sites ethical approval was granted for confidential abstraction of data from archived case records without individuals’ consent due to the retrospective design. The Kenya Medical Research Institute/National Ethical Review Committee approved the study.

### Study Subjects

We obtained data on paediatric admissions and outcomes in nine Kenyan District hospitals. In one hospital data were collected as part of ongoing, prospective clinical surveillance (Kilifi District Hospital, H9) and in eight sites (H1 to H8) data were obtained during conduct of a multicenter trial aimed at improving quality of inpatient paediatric care (described in full elsewhere [8]). All admissions to the nine participating hospitals H1–H9, aged 2 to 59 months admitted with acute illnesses from March 2007 to March 2008 (inclusive) were eligible for inclusion in the analysis. A random sample was drawn from these eligible admissions in eight hospitals (H1–H8) while in H9 all eligible children were included in the study. The sampling in the eight hospitals (H1–H8) was based on randomly selecting calendar dates from two consecutive 6-month periods in each hospital. All admissions on the sampled dates were included in the study using each hospital’s admission rate to adjust the proportion of selected dates to yield approximately 400 medical records per hospital during each 6-month period (Table 1). For this particular analysis the inclusion criteria was admission with any pneumonia diagnosis.

**Table 1:** Admissions aged 2–59 months at 9 Kenyan District Hospitals from March 2007 to March 2008 and characteristics of those with pneumonia diagnosis.

	H1	H2	H3	H4	H5	H6	H7	H8	H9	Overall
Number of admissions	515	555	537	518	501	363	558	507	2547	6601
Admissions with any pneumonia diagnosis, n*(%)	211(41.0)	378(68.1)	241(44.9)	285(55.0)	111(22.2)	195(53.7)	244(43.7)	264(52.1)	1443(56.7)	3372(51.1)
Pneumonia	55(26.1)	76(20.1)	89(36.9)	94(33.0)	9(8.1)	24(12.3)	37(15.2)	51(19.3)	562(38.9)	997(29.6)
Severe pneumonia	111(52.6)	192(50.8)	107(44.4)	145(50.9)	12(10.8)	91(46.7)	84(34.4)	154(58.3)	612(42.4)	1508(44.7)
Very severe pneumonia	35(16.6)	100(26.5)	39(16.2)	35(12.3)	-	11(5.6)	18(7.4)	37(14.0)	269(18.6)	544(16.1)
No classification†	10(4.7)	10(2.6)	6(2.5)	11(3.9)	90(81.1)	69(35.4)	105(43.0)	22(8.3)	-	323(9.6)
Median age in months (IQR)	14(7–25	11(7–20)	12(7–24)	12(7–24)	14.4(8–28)	10(6–17)	12(7–24)	11(6–20)	16(8.3–29)	12(6–23)
Male	103(48.8)	213(56.4)	127(52.7)	138(48.4)	57(51.4)	95(48.7)	108(44.6)	138(52.3)	814(56.4)	1793(53.2)

* n represents denominator for the pneumonia classifications and child’s sex presented below.

† represents a pneumonia diagnosis in which the clinician failed to classify severity as pneumonia, severe pneumonia or very severe pneumonia.

H9 is Kilifi District hospital where data is collected in a prospective on-going surveillance of all inpatients.

### Study Sites

#### Cross-sectional data

Data from the multicenter study were obtained from 8 rural district hospitals (H1 to H8) from four of Kenya’s eight provinces. The characteristics of these hospitals and the interventions received have been described previously. [8], [9] In brief these eight hospitals spanned highland and semi-arid areas with low risk of malaria transmission and areas near Lake Victoria with moderate to high risk of malaria transmission. Sites also varied in estimated ante-natal HIV prevalence (from <5% to >10% in 2005), estimated infant mortality rate (approximately 40 to over 100 per 1,000 in 2005) and hospital size (996 to 4738 paediatric admissions in 2005). [8] Data from the study used in the current analysis were collected from these sites and from Kilifi District Hospital (described below) during the first 12 months (March 2007 to March 2008) of the 18 month intervention period when all sites were expected to use national pneumonia case management guidelines.

#### Kilifi District hospital

Kilifi District Hospital (KDH) is located in a rural area along the Kenyan coast with clinical and laboratory surveillance undertaken on all pediatric admissions since 1998. [10] The hospital serves a predominantly poor population and has a research center -Kenya Medical Research Institute’s Center for Geographic Medicine Research (CGMRC) - located within it. Approximately 5000 children are admitted annually to the 42-bed paediatric ward. [11] The hospital is located in a malaria endemic area and the current antenatal HIV prevalence is approximately 4.7%. [12] All paediatric admission episodes to KDH are now prospectively documented using an electronic medical record which contains information on clinical assessment, treatment and discharge outcomes. As part of the multi-centre study a paper paediatric admission record was introduced into the eight hospitals described above to capture similar information to that available from the electronic medical record at KDH.

#### Data quality

Missing data is commonly encountered within data sets collected in routine clinical settings as in our study. This is less of a problem in settings with an electronic medical record system such as Kilifi District Hospital (H9). During this study we attempted to improve quality of routine data from the eight remaining hospitals through introducing structured paediatric admission records (PAR) for documenting childhood admission episodes in hospitals (H1–H8). Details of the development, piloting and introduction of the PAR have been presented previously. [8], [13] Although the tool was widely accepted by clinicians and greatly improved documentation of childhood illnesses in Kenyan District Hospital settings there were disparities in PAR use among clinicians and across hospitals explaining the differences in completeness of documentation of clinical features of illness in data collected in the eight hospitals- H1 to H8.

#### Study outcome

The main outcome in this analysis was pneumonia mortality. The pneumonia classification adopted for our analysis was based on WHO pneumonia guidelines (Box S1). Paediatric clinical health workers within each hospital were trained to apply pneumonia severity classification in diagnosis and management of acute respiratory illness as outlined in national guidelines adapted from WHO recommendations. The primary cause of death was assigned at the analysis stage. Admissions with severe malnutrition (visible wasting) and/or meningitis were excluded from analysis because these two conditions are more likely to be assigned as primary causes of death even in the presence of pneumonia. Where children presented with both non severe pneumonia, a condition with very low mortality in the presence of biomedical treatment, and malaria or diarrhea and dehydration the latter diagnosis was assigned as the primary cause of death. Otherwise, for children with a diagnosis of severe or very severe pneumonia the presenting pneumonia diagnosis was considered the primary cause of inpatient death. The primary cause of death could not be determined in sixteen children with unclassified pneumonia who were therefore excluded from the pneumonia mortality analysis.

#### Cross-sectional analysis of pneumonia mortality

Cross-sectional data collected on admission and at death or discharge from admissions to all nine hospitals between April 2007 and March 2008 were used for this analysis. We determined that the data available for our analysis would allow us to estimate pneumonia case fatality within each hospital with adequate precision for both hospitals reporting high and low mortality rates. For hospitals with the lowest pneumonia case fatality (approximately 4%) we required at least 369 observations per site to estimate mortality with a precision of ±2%. On the other hand, in hospitals with mortality rates of 10% or above at least 138 observations per hospital would allow estimation of inpatient pneumonia case fatality with precision of ±5%.

Co-morbid illnesses including clinical diagnoses of malaria and diarrhoea and/or dehydration, age and gender of child were obtained from the medical records and all these data were merged with the discharge vital statistic information, indicating whether a child died during admission or was discharged alive. Patient level data on HIV status were not routinely available. Data cleaning and analysis was conducted using STATA version 11 (StataCorp, Tx USA). The distribution of pneumonia cases by reported illness severity, age, gender and common co-morbidities was described within each hospital. Crude and adjusted case fatality rates for pneumonia were estimated within each hospital. To do this a binomial regression model with a logit link, including a fixed effect for hospitals, was used to estimate odds ratios for pneumonia mortality within each hospital adjusted for age, gender, and malaria or diarrhea comorbidity. Predicted probabilities representing average pneumonia case fatality in each hospital were obtained from the regression model. The assessment of significant variation in pneumonia case fatality was conducted using likelihood ratio tests.

We conducted a sensitivity analysis to assess the impact of including H9 in the analysis for two reasons. First, unlike the other hospitals H9 has a research center based within it and this center has been conducting research on common childhood illnesses within the hospital’s paediatric unit since 1989. The quality of data available and possibly quality of care in this hospital therefore differed from that in the remaining District Hospitals. Second, this hospital contributed around 40% of the total observations in the final regression model used to estimate adjusted pneumonia mortality and associated variations in mortality. We employed user defined weighting in the regression analysis for data from H9 and conducted regressions using five different increasing weights: 0 (equivalent to exclusion of KDH data from analysis), 0.3, 0.5 and 0.8. These regressions were compared to the base case (equally weighted) analysis which was equivalent to a weight of 1.0.

### Time Series Analysis of Trends in Pneumonia Mortality

Data collected from January 1999 to March 2008 at KDH were aggregated by time point and analysed by specifying a segmented linear regression model with the mean monthly pneumonia case fatality rate as the unit of analysis. Pneumonia mortality trends during the period were examined using interrupted time series to explore the potential impact of nationwide Hib vaccine introduction in November 2001 on pneumonia mortality. To account for the lag in vaccine effect our segmented regression model excluded the year of Hib introduction and one year post-vaccine introduction (November 2001–November 2003).

In addition to the outcome of pneumonia mortality, the regression model included terms for Hib vaccine introduction, and secular trends for the time periods before and after Hib introduction. The resulting model coefficients represented pneumonia mortality trends in the two analysis periods and the change in level of mortality post Hib introduction based on the approach proposed by Wagner and colleagues. WHO case definition of pneumonia, severe pneumonia and very severe pneumonia for children aged 2 to 59 months.

Serial autocorrelation between the error terms for case fatality rates over time was tested using the Durbin-Watson statistic and the model controlled for autocorrelation by correcting for first order autoregressive effects. Seasonal variations in pneumonia mortality were demonstrated, with higher case fatality rates occurring from April to June. These seasonal effects were accounted for by including indicator terms representing each calendar month in the regression model.

## Results

Cross-sectional data collected from all nine hospitals were available for 3 372 pneumonia admissions from March 2007 to March 2008 (1 443 from KDH and 1 929 from the remaining 8 hospitals). There were an additional 15 739 pneumonia cases drawn from 28 160 admissions to KDH between January 1999 and February 2007 and used for the time series analysis presented at the end of this results section.

### Participants’ Characteristics

In the one-year cross-sectional mortality analysis, approximately 50% of the patients admitted across hospitals were male (Table 1). Overall, the median age of pneumonia admissions was 12 months (interquartile range 6 to 23) varying across the hospitals from 8 to 13.5 months (Kruskal-Wallis χ^2^, 8 d.f = 38.1, p = 0.0001). In the time series data for children admitted to KDH (H9) patient characteristics did not change substantially between January 1999 and February 2007 (data not shown) or differ significantly from that of children admitted in 2007–2008 shown in Table 1.

### a) Cross-sectional Analysis

#### Pneumonia case management

Approximately 90% of 3372 children with a pneumonia diagnosis across all hospitals in 2007–2008 had a severity classification based on WHO recommendations. Very severe pneumonia accounted for 544 (16.1%) cases, severe pneumonia for 1508 (44.7%) and pneumonia accounted for 997 (29.6%) of all pneumonia cases (Table 1). The remaining 323 (9.6%) children were not assigned a severity classification for pneumonia. Failure to assign a severity classification varied across hospitals and was observed in between 35% and 81% of admissions to H5, H6 and H7, and less than 10% of admissions to H1 to H4 and H8. All children admitted with pneumonia to H9 were assigned a severity classification (Table 1).

#### Inpatient pneumonia mortality

Among the 3372 children admitted with pneumonia from March 2007–March 2008 no information was available on hospitalization outcome of 53 (1.6%) children. Of the remaining 3 319 children with pneumonia 195 (5.9%) children died. The overall case fatality rates for pneumonia, severe pneumonia and very severe pneumonia were 19 out of 997 (1.91%), 72 out of 1508 (4.77%) and 82 out of 544 (15.07%), respectively. The overall risk of death among children admitted with very severe pneumonia was significantly higher than for severe pneumonia (15.07% versus 4.77%, risk ratio = 3.13, 95%CI 2.32 to 4.24, p<0.001).

The unadjusted overall pneumonia case fatality rates in the nine hospitals ranged from 3.7% in H2 to 13.9% in H5 (Table 2). After adjusting for the effect of age, gender, and common co-morbidities including malaria and diarrhoea/dehydration the case fatality rates varied significantly between hospitals (LR χ^2^ = 52.19; p<0.001), ranging from 3.1% (95%CI, 0.9 to 5.4) in H7 to 13.2% (95%CI, 6.9 to 19.5) in H5 (Table 2).

**Table 2:** Outcome for paediatric admissions with pneumonia in 9 Kenyan district hospitals.

	Pneumonia deaths (CFR)				UnadjustedCFR (95%CI)	Adjusted CFR †(95% CI)
Hospital	Pneumonia	Severepneumonia	Very severe pneumonia	No classification	Overall	Overall
H1	1(1.9)	10(9.7)	9(26.5)	1(14.3)	10.7(6.7,15.8)	9.5(5.5,13.4)
H2	1(1.3)	6(3.1)	7(7.0)	0	3.7(2.0,6.2)	3.9(1.9,5.8)
H3	3(3.5)	4(3.8)	6(15.4)	0	5.5(3.0,9.2)	5.6(2.6,8.5)
H4	2(2.1)	21(14.6)	9(25.7)	1(9.1)	11.6(8.1,15.9)	12.1(8.4,15.8)
H5	1(12.5)	1(8.3)	0	13(14.8)	13.9(8.0,15.9)	13.2(6.9,19.5)
H6	1(8.7)	4(4.9)	1(9.1)	4(6.3)	6.1(3.1,10.7)	6.6(2.9,10.3)
H7	0	3(3.7)	3(17.7)	1(0.97)	3.0(1.2,6.0)	3.1(0.9,5.4)
H8	4(7.8)	11(7.2)	6(16.2)	2(9.1)	8.8(5.6,12.8)	8.7(5.3,12.1)
H9	5(0.89)	12(2)	41(15.4)	0	4.0(3.1,5.2)	4.0(3.0,5.0)

* Case fatality rate.

† Case fatality rate adjusted for patient’s age, gender, malaria & diarrhoea/dehydration comorbidity.

In the adjusted analysis, age, diarrhea co-morbidity and pneumonia severity showed statistically significant associations with pneumonia mortality (Table 3). Compared to infants (2 to 11 months) with a case fatality rate of 8.8%, the odds of mortality were approximately 60% lower for children aged 12–24 months (3.5%) and 25–59 months (3.3%), p<0.001. A co-morbid diarrhea diagnosis was independently associated with higher fatality in the adjusted analysis (Odds Ratio = 1.50, 95% CI 1.04–2.15, p = 0.028, Table 3). Increasing pneumonia severity was also associated with higher case fatality rates. Compared to severe pneumonia admissions with children with very severe pneumonia had the highest risk of death (OR 9.17, 95% CI 5.37–15.67) followed by severe pneumonia (OR 2.22, 95% CI 1.30–3.79).

**Table 3:** Effect of age, sex, and comorbidity on pneumonia mortality adjusted for hospital.

	Died (%)	Survived (%)	Unadjusted OR (95% CI)	Adjusted OR (95% CI)
Age group				
2–11 months	135 (8.8)	1400 (91.2)	1.00	1.00
12–24 months	34 (3.5)	952 (96.5)	0.41 (0.31–0.54)	0.39 (0.25–0.58)
25–59 months	26 (3.3)	772 (96.7)	0.42 (0.32–0.56)	0.42 (0.27–0.67)
Sex				
Female	90 (6.2)	1353 (93.8)	1.00	1.00
Male	97 (5.5)	1665 (94.5)	0.89 (0.74–1.12)	0.87 (0.64–1.19)
Pneumonia severity				
Pneumonia	19 (9.1)	966 (98.1)	1.00	1.00
Severe pneumonia	72 (4.9)	1412 (95.1)	2.59 (1.55–4.33)	
Very severe pneumonia	82 (15.2)	457 (84.8)	9.12 (5.47–15.21)	9.17 (5.37–15.67)
Pneumonia without severity classification	22 (7.1)	289 (92.9)	3.81 (2.06–7.25)	2.54 (1.12–5.80)
Malaria				
No malaria	170 (6.0)	2666 (94)	1.00	1.00
Moderate malaria	4 (1.9)	202 (98.1)	0.31 (0.11–0.85)	0.47 (0.16–1.36)
Severe malaria	18 (9.1)	180 (90.9)	1.57 (0.94–2.61)	1.15 (0.65–2.04)
Malaria without severity classification	3 (3.8)	76 (96.2)	0.62 (0.19–1.98)	0.44 (0.12–1.55)
Diarrhea/dehydration				
No	135 (5.1)	2543 (94.9)	1.00	1.00
Yes	60 (9.2)	590 (90.8)	1.47 (1.17–1.86)	1.50 (1.04–2.15)

#### Sensitivity analysis

Inclusion of data from H9 did not change the direction of any significant effect for the covariates used to explain pneumonia mortality in the fixed effects model. (Table S1) Regardless of the weight assigned to hospital H9 data, the magnitude of effect for age (12–24 months) on mortality was consistent as was the effect of severe pneumonia classification. The effect of age (25–29 months), very severe pneumonia classification and malaria co-morbidity increased progressively with increasing importance weight of H9 data.

### b) Time Series Analysis of Pneumonia Mortality at KDH

**Figure 1:**
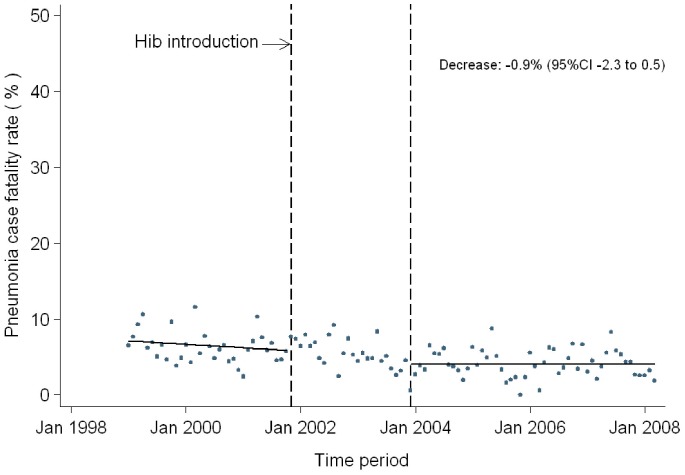
Interrupted time series analysis of pneumonia mortality among paediatric admissions to Kilifi District Hospital (1999 to 2008).

The data available from Kilifi District Hospital covered a period of 9.25 years representing 111 monthly time points. During this period a total of 17 179 admissions were diagnosed with pneumonia and the average monthly mortality rate was 6.6% over the entire period. In January 1999, the estimated mortality rate was 6.0% (95% CI, 3.6 to 8.4%). The trend in pneumonia mortality remained constant between 1999 and November 2001 (trend −0.03, 95% CI −0.1 to 0.02, p = 0.36) when the Hib vaccine was introduced (Figure 1). There was a statistically insignificant decline in pneumonia case fatality rate (−0.9, 95% CI −3.9 to 2.1, p = 0.55) at the end of 2003, two years after Hib introduction. No significant trend in pneumonia deaths was seen between November 2003 and the end of the study (trend 0.04, 95% CI −0.04 to 0.11, p = 0.35).

## Discussion

The high mortality attributed to pneumonia in childhood has been reported in numerous publications, [14], [15] but surprisingly there is very little systematic analysis of pneumonia deaths in hospital in developing countries. This study is among the first formal multicenter comparisons of inpatient pneumonia mortality undertaken in the Hib vaccine era in Africa and similar low income settings. Importantly, the study explores temporal changes in one site as a possible explanation to the observed variation in hospital mortality. Our study demonstrated significant variations in inpatient paediatric pneumonia mortality rates, with the adjusted mortality rates varying by up to three-fold across hospitals. Although we could examine only one site we found considerable consistency in mortality rates over a period spanning 9.25 years suggesting that temporal variations may not explain the observed cross-sectional variation in inpatient pneumonia mortality.

What might explain variability in mortality? Several risk factors for mortality, including co-existent malnutrition or HIV, could explain the observed between hospital variations. A clear impact of HIV on childhood mortality in sub-Saharan Africa has become apparent in recent years especially in southern and eastern African countries, [16] and malnutrition is considered a contributory factor in many childhood deaths. [17] Although children also diagnosed with severe malnutrition were excluded from the study it is possible that prevalence of lesser degrees of malnutrition that could not be identified in our study will vary and influence case fatality rates. Unfortunately individual patient HIV information was not routinely collected during the study period. Estimates of antenatal HIV prevalence in areas served by hospitals were available but were not used in our analysis because such hospital-level effects are effectively captured in the hospital estimates derived from fixed effects models. Conversely, it is noteworthy that in a rural Zambian hospital pneumonia mortality was not significantly affected by HIV infection in children presenting with and appropriately treated for acute lower respiratory infection. [18].

Our findings from analysis of time series data from Kilifi confirm the seasonality of pneumonia mortality in Kilifi agreeing with previously demonstrated seasonal patterns for LRTI incidence within the same setting. [19] After accounting for the seasonality in pneumonia case fatality, data from Kilifi demonstrated a non-significant decline in pneumonia mortality post-Hib introduction but no evidence of an overall trend in pneumonia mortality over a 9.25-year period. Cowgill *et al* reported significant reductions in the incidence of invasive *Haemophilus influenzae* disease, a major cause of bacterial pneumonia, in Kilifi district in 2004, three years after introduction of routine Hib vaccination. [11] Pneumonia case fatality rates remained relatively stable despite some evidence for a decline in HIV prevalence over this period. [12] In the multivariate analysis, we found that the odds of pneumonia deaths outside the infant age group was approximately half that of infants. Similar declines in risk of pneumonia mortality with increasing age have been reported previously. [14].

While interpreting the interrupted time series analysis in our study it is important to consider several factors. First, the primary aim of this analysis was not to determine effectiveness of Hib vaccine in preventing pneumonia mortality (i.e. change in level of the two segments of the time series). Instead the analysis intended to determine pneumonia mortality trend (i.e. slopes of the two segments) among children hospitalized with severe pneumonia while accounting for vaccine introduction during the analysis period. Demonstrating vaccine effectiveness would require incorporating outpatient pneumonia incidence and outcomes data and is best implemented using community based surveillance studies. Second, we considered possible alternative explanations for the lack of a significant vaccine effect on severe inpatient pneumonia mortality including low vaccination coverage. The estimated vaccine coverage within Kilifi district at the point of vaccine introduction was relatively high- 87% at 12 months of age. (Cowgill) This coverage reduces after the infancy period but the average national coverage at 12–23 months of age during 2008/09 was 77%. (KDHS).

Our study applied the WHO definition of severe pneumonia which captures a broad group of critically ill children with respiratory distress, including metabolic acidosis, lowering its sensitivity. We observed a variable effect of co-morbid illnesses on pneumonia mortality. While children with diarrhea comorbidity had a higher risk of mortality admissions with malaria comorbidity did not. Interestingly over the period spanning deployment of Hib vaccine and declining HIV prevalence malaria prevalence has also declined. The consistency of pneumonia case fatality rates may thus represent the net effect of multiple factors that we are poorly placed to understand as surveillance data are inadequate.

Another key requirement in understanding pneumonia mortality and its variability is potential variation in case-severity. Variation in patterns of illness severity at presentation may reflect the prevalence of underlying, biological risk factors in a hospital’s catchment area but may also capture social, economic, cultural, and geographic influences on access to services. While there was clear evidence that children classified as more severely ill had higher mortality in general failure to classify patients and likely poor standardization of classification currently make it hard to use data on case-severity to explain variability in overall inpatient pneumonia mortality rates.

The interpretation and use of hospital mortality or cause specific mortality rates to understand health system performance have been widely debated in the literature. [4] However, there seems little doubt that we should have access to such basic data. At present, as in Kenya, this is rarely the case in low income settings. The data we present serve to demonstrate that variability can be marked across place while there was less evidence for variation across time (although data are very limited). Understanding this variation could help identify location-specific priorities for intervention and help in assessing efforts to strengthen health systems. Unfortunately the current scarcity of data reflect inadequate attention to supporting development of robust, routine information systems and, we suggest, the result is data that are inadequate for informed global or national decision making.

### Study Strengths and Limitations

Our study compares case fatalities in nine facilities in different settings and assesses the impact of some important covariates of mortality. This overcomes the limitation of previous estimates from single hospitals that did not describe risk factors of mortality. The data used in this study is based on data of reasonable quantity and quality in nine sites where staff training in use of guidelines and structured paediatric admission records were available to facilitate improved data collection.

However, the findings we report are subject to several limitations. In common with all studies conducted using routinely collected data it is difficult to ascertain that a specific or rigorous case definition for pneumonia was applied by health workers in routine settings. Separately, failure to classify pneumonia severity occurred in approximately one in every ten admissions and was more common in three out of the nine hospitals introducing possible bias in the study. Between one-third and 81% of patients in these three hospitals lacked a classification, resulting in weaker associations than would be reported if all children had a pneumonia severity classification. Furthermore data on individuals’ HIV status were not available. Such problems could be overcome by improving approaches to standardising clinical assessment, documentation and treatment which have already been explored by other studies in our setting. [13], [15] Additional bias could result from the restricted geographical sampling and small number of facilities in the study in relation to the total number of similar facilities in the country. However, our purpose was not to suggest generalisabilty of a specific finding but more to illustrate the potential for variation.

The modelling approach, limited by data availability and the number of hospitals studied, captures multiple possible factors that may contribute to observed variability within the fixed term for hospital. Determining which factors are actually important determinants of variability would require a considerably larger dataset. Lastly, and of particular relevance to the time series analysis is the fact that our study was not adequately powered or designed to detect the impact of routine Hib vaccination on mortality. To demonstrate such an effect, large carefully designed studies similar to the assessment of the impact of pneumococcal vaccination on invasive pneumococcal disease conducted in the United States using population data from several states need to be conducted. [20].

### Policy Implications and Future Analyses

Despite these limitations the findings of this study are useful The estimates we report can find application in several areas of Decision Analysis: (a) as case fatality estimates for modelling effects of interventions in similar settings where such data is unavailable, (b) to help define plausible case fatality ranges in sensitivity analyses, and (c) to address model structure uncertainty when examining previous models that used simplifying assumptions about case fatality. In the longer term such models would benefit from systems in place at regional or national levels to collect basic clinical and epidemiological data for use in such analyses. [21] The study, by demonstrating marked variability in pneumonia fatality, provides a clear challenge to all to explain this. From a health systems perspective key questions are whether variability in quality of care, recently identified in a number of studies, or socio-economic or biological factors are major explanatory factors. Addressing such questions and evaluating whether interventions are having an impact will clearly require much better data at greater scale. Lastly, long term follow-up studies of post discharge pneumonia mortality are needed but such analysis could not have been implemented in this study focusing on inpatient outcomes.

## Supporting Information

Table S1 **Sensitivity analysis of the impact of different user defined weights for H9 on model estimates of effect of age, sex, and comorbidity on pneumonia mortality.**
(DOC)

Box S1 **WHO case definition of pneumonia, severe pneumonia and very severe pneumonia for children aged 2 to 59 months.** *Nasal flaring, grunting, indrawing, raised RR
(DOC)
