# Supplementary material for: Variations in Mortality in Children Admitted with Pneumonia to Kenyan Hospitals
Source: PLoS One. 2012 Nov 5;7(11):e47622. doi: 10.1371/journal.pone.0047622 (PMC3489903; doi:10.1371/journal.pone.0047622)
Supplement: Table S1 — Sensitivity analysis of the impact of different user defined weights for H9 on model estimates of effect of age, sex, and comorbidity on pneumonia mortality. (DOC) [file pone.0047622.s001.doc]

**Table S1:** Sensitivity analysis of the impact of different user defined weights for H9 on model estimates of effect of age, sex, and comorbidity on pneumonia mortality

| **H9 weight** | **Model 1**  **0** | **Model 2**  **0.3** | **Model 3**  **0.5** | **Model 4**  **0.8** |
| --- | --- | --- | --- | --- |
|  | **Adjusted OR**  **(95% CI)** | **Adjusted OR**  **(95% CI)** | **Adjusted OR**  **(95% CI)** | **Adjusted OR**  **(95% CI)** |
| **Age group** |  |  |  |  |
| 2-11 months | 1.00 | 1.00 | 1.00 | 1.00 |
| 12-24 months | 0.41 (0.25-0.66) | 0.40 (0.25-0.63) | 0.40 (0.25-0.62) | 0.40 (0.25-0.60) |
| 25-59 months | 0.18 (0.08-0.38) | 0.27 (0.15-0.49) | 0.32 (0.19-0.55) | 0.39 (0.24-0.63) |
| **Sex** |  |  |  |  |
| Female | 1.00 | 1.00 | 1.00 | 1.00 |
| Male | 1.14 (0.78-1.66) | 1.02 (0.72-1.46) | 0.97 (0.69-1.36) | 0.91 (0.66-1.25) |
| **Pneumonia severity** |  |  |  |  |
| Pneumonia | 1.00 | 1.00 | 1.00 | 1.00 |
| Severe pneumonia | 2.27 (1.21-4.26) | 2.25 (1.24-4.08) | 2.24 (1.26-3.98) | 2.23 (1.28-3.86) |
| Very severe pneumonia | 5.99 (3.04-11.81) | 7.18(3.86-13.36) | 7.84(4.34-14.17) | 8.69 (4.99-15.14) |
| Pneumonia without severity classification | 2.35 (0.98-5.62) | 2.54 (1.03-5.70) | 2.47 (1.06-5.74) | 2.52 (1.10-5.78) |
| **Malaria** |  |  |  |  |
| No malaria | 1.00 | 1.00 | 1.00 | 1.00 |
| Moderate malaria | 0.23 (0.05-1.01) | 0.31 (0.09-1.14) | 0.36 (0.11-1.21) | 0.40 (0.14-1.31) |
| Severe malaria | 0.76 (0.38-1.50) | 0.90 (0.47-1.72) | 0.98 (0.53-1.83) | 1.09 (0.61-1.97) |
| Malaria without severity classification | 0.44 (0.12-1.57) | 0.44 (0.12-1.56) | 0.44 (0.12-1.56) | 0.44 (0.12-1.55) |
| **Diarrhea/dehydration** |  |  |  |  |
| No | 1.00 | 1.00 | 1.00 | 1.00 |
| Yes | 1.76 (1.16-2.66) | 1.67 (1.13-2.47) | 1.61 (1.10-2.36) | 1.54 (1.06-2.22) |
