# Supplementary material for: Variations in Mortality in Children Admitted with Pneumonia to Kenyan Hospitals
Source: PLoS One. 2012 Nov 5;7(11):e47622. doi: 10.1371/journal.pone.0047622 (PMC3489903; doi:10.1371/journal.pone.0047622)
Supplement: Box S1 — WHO case definition of pneumonia, severe pneumonia and very severe pneumonia for children aged 2 to 59 months. *Nasal flaring, grunting, indrawing, raised RR (DOC) [file pone.0047622.s002.doc]

**BOX S1:** WHO case definition of pneumonia, severe pneumonia and very severe pneumonia for children aged 2 to 59 months

*Entry criteria for a pneumonia diagnosis:*

-History of cough or difficult breathing

*Pneumonia:*

-Raised respiratory rates (age-specific)

**-**Age 2 – 11 months: Respiratory rate ≥ 50

-Age ≥12 months: Respiratory rate ≥ 40

*Severe pneumonia*

-Lower chest wall indrawing

*Very severe pneumonia*

- Cyanosis, inability to drink / breast feed, or altered consciousness, (often + other respiratory signs*)
